# Nursing & parental perceptions of neonatal care in Central Vietnam: a longitudinal qualitative study

**DOI:** 10.1186/s12887-017-0909-6

**Published:** 2017-07-11

**Authors:** Katie Gallagher, Colin Partridge, Hoang T Tran, Suzanna Lubran, Duncan Macrae

**Affiliations:** 1 Institute for Women’s Health, University College London, 74 Huntley Street, London, WC1E 6AU UK; 2 Department of Pediatrics, University of California, San Francisco, USA; 3 Neonatal Unit, Da Nang Hospital for Women and Children, Da Nang, Vietnam; 4 Newborns Vietnam, Da Nang, Vietnam, http://www.newbornsvietnam.org; 5 Pediatric Intensive Care, Royal Brompton and Harefield NHS Trust, London, UK

**Keywords:** Neonatal intensive care, Nursing care, Parent experience, Developing countries

## Abstract

**Background:**

Neonatal mortality accounts for nearly three quarters of all infant deaths in Vietnam. The nursing team are the largest professional group working with newborns, however do not routinely receive neonatal training and there is a lack of research into the impact of educational provision. This study explored changes in nursing perceptions towards their role following a neonatal educational intervention. Parents perceptions of nursing care were explored to determine any changes as nurses gained more experience.

**Method:**

Semi-Structured qualitative interviews were conducted every 6 months over an 18 month period with 16 nurses. At each time point, parents whose infant was resident on the neonatal unit were invited to participate in an interview to explore their experiences of nursing care. A total of 67 parents participated over 18 months. Interviews were conducted and transcribed in Vietnamese before translation into English for manifest content analysis facilitated by NVivo V14.

**Results:**

Analysis of nursing transcripts identified 14 basic categories which could be grouped (23) into 3 themes: (1) perceptions of the role of the neonatal nurse, (2) perception of the parental role and (3) professional recollections. Analysis of parent transcripts identified 14 basic categories which could be grouped into 3 themes: (1) information sharing, (2) participation in care, and (3) personal experience.

**Conclusions:**

Qualitative interviews highlighted the short term effect that the introduction of an educational intervention can have on both nursing attitudes towards and parental experience of care in one neonatal unit in central Vietnam. Nurses shared a growing awareness of their role along with its ethical issues and challenges, whilst parents discussed their overall desire for more participation in their infants care. Further research is required to determine the long term impact of the intervention, the ability of nurses to translate knowledge into clinical practice through assessment of nursing knowledge and competence, and the impact and needs of parents. A greater understanding will allow us to continue to improve the experiences of nurses and parents, and highlight how these areas may contribute towards the reduction of infant mortality and morbidity in Vietnam.

**Electronic supplementary material:**

The online version of this article (doi:10.1186/s12887-017-0909-6) contains supplementary material, which is available to authorized users.

## Background

In Vietnam significant progress has been made towards the achievement of reducing the under 5 mortality rate in line with the UN Millennium Development Goals (MDG 4) and the subsequent Sustainable Development Goals (Goal 3), both seeking to eradicate health inequalities and strengthen country progression though social, economical and environmental targets [1, 2]. Despite being ranked as a ‘poor’ country by the Human Development Index (116/188) [3], Vietnam’s health indicators are better than expected for a country with low income per capita [2]. Since 1990, the under 5 mortality rate in Vietnam has declined from 58 per 1000 live births to 24 per 1000 [4]. In contrast, however, neonatal mortality has decreased slowly throughout this time and still accounts for nearly three-quarters of all infant deaths [5], with large variation seen between different districts within the same province [6]. Given that infant mortality remains a significant public health issue in Vietnam, newborn care has undergone rapid expansion during the past decade [4]. All provincial hospitals now have neonatal units, although with varying resources [7]. To achieve further progress in this area, however, the knowledge, skills and systems of the neonatal workforce need to be understood, as translation and implementation of appropriate knowledge into practice remains a global health problem [6]. This is particularly significant in low and middle income settings where there is little evidence to support the notion that practices which work well in one country will work well in another [8]. Empowering the local health care workforce through knowledge and skills training is essential to ensure relevance and feasibility of the training process. In neonatal care, the nursing team play a major role in the delivery of care, constituting the largest professional group amongst the health care workforce working with newborns [9]. Nursing staff have the greatest contact with patients and their families, therefore having the greatest potential for preventative education to reduce infant mortality [9].

Nursing in Vietnam has made many advances in the past decade, including a growing recognition of an expanded nursing role following a name change from ‘Implementer of Doctors Order’ (‘Y tá’) to ‘nurse’ (‘diệu dương’) [10]. There remain significant issues however, despite the development of a national examination following graduation [9], as there are shortages of qualified faculty, a lack of teaching resources and no standardised nursing procedures [11]. There is a lack of research into the impact of nursing provision and education in Vietnam in all areas, but in particular in neonatal nursing where staff are often sent from other specialities within the hospital and receive no specific neonatal training. Without specialist knowledge in newborn care, further reductions in neonatal mortality have been hard to achieve. To address this, funding was successfully sought in 2013 to implement a structured neonatal nursing programme over an 18 month period in one pilot hospital in Vietnam. The programme, led and coordinated by Canterbury Christ Church University (CCCU), was implemented on a bi-monthly basis with experienced neonatal nurse lecturers from the UK teaching classes covering aspects of neonatal care such as thermoregulation, respiratory assessment and developmental care. Following completion of the education programme, we explored whether there were any changes in the attitudes and perceptions of the neonatal nurse participants towards their role and the role of the parents of the infants with whom they worked, at defined time points over 18 months. We also explored the parents’ perceptions to determine any changes in family experience as the nurses gained experience. This paper focuses specifically on the outcomes of this research, the first of its kind in Vietnam.

## Methods

### Aim

The aim of this study was to explore changes in the perceptions and attitudes of nurses and parents towards their experiences in the neonatal unit following a neonatal nursing education intervention in a single neonatal unit in central Vietnam. Details of the course development, structure and evaluation can be found in West et al. (unpublished data) [12].

### Design

We chose a qualitative longitudinal research design to capture changes in perceptions through interviews conducted at different time points following the completion of the training programme, providing insight into the phenomena as it evolved through time [13]. The study team consisted of specifically trained local, independent researchers, under the supervision of the lead principle investigator on the neonatal unit. Guidance and support was provided by a multidisciplinary research team based in the UK and the USA. A semi-structured interview schedule was developed following a review of the educational content of the training course and current literature, exploring nursing participants views of their role as a neonatal nurse within the context of both the professional team and the families with whom they work, and their perceptions of training. For parents, content explored their perceived involvement in their infants care and their experiences on the neonatal unit. Content validity of the interview schedules were provided through expert panel review by experienced researchers in the neonatal field, with feasibility tested through a pilot interview with one of the leading neonatologists on the study unit to ensure cultural acceptability of the questions and minimise any errors in translation. The study included 2 groups of participants: parents of infants admitted to the neonatal unit and neonatal nurses who had undertaken the education intervention. Different parents were recruited at each time point, whilst the same nurses were interviewed at 6 monthly intervals between 2014 and 2015 on one neonatal unit in Vietnam. Ethical approval was granted by the Scientific and Ethics Committee of the Da Hang Hospital for Women and Children.

### Recruitment

Recruitment took place in neonatal unit in the Da Hang Hospital for Women and Children Vietnam, the largest level neonatal III unit in central Vietnam with an 80-cot capacity providing both medical and surgical care to newborn infants from 25 weeks’ gestation. There are around 14,000 live births each year. Up to 40% of admissions (both transferred in utero and neonatal period) come from surrounding provinces unable to care for very sick neonates, creating an approximate catchment area of 4 million people. Over the course of one week at three different consecutive time points (T1, T2, T3) parents from all areas of the neonatal unit (special care, high dependency and intensive care) were approached and informed about the study by a group of independent specially trained Vietnamese researchers. Parent participants were invited to participate in a semi-structured interview exploring their attitudes and experiences towards neonatal care. During the first time point, 21 parents volunteered to participate in the interviews. To determine whether saturation had been reached (and therefore whether more parents were required), initial analysis of the data was undertaken. With the emergence of no new codes at this point it was felt the sample size was appropriate. The aim was therefore to recruit a similar number of parents at T2 and T3, where 23 parents respectively volunteered to participate. All neonatal nurses (*n* = 26) who had completed the training were invited to participate for the duration of the study, involving 3 interviews at 6 monthly intervals over the course of 18 months. Of the 26 nurses, 16 volunteered to participate. All potential participants were informed of the aim of the study and given time to consider volunteering, and enrolled if they wished to participate. All signed informed consent prior to the study.

### Data collection

The first phase of data collection was conducted in August 2014 following the completion of the neonatal nursing educational intervention in spring 2014. Two further phases of interviews were conducted at 6 monthly interviews. The same group of researchers conducted the interviews at each time point using the same semi structured interview questions. Interview schedules for both nurses and parents are available as Additional files 1 and 2 with this paper. Interviews took around 20 min to complete and were scheduled over the course of a week at each time point. Both parent and nursing interviews were audio recorded and took place in a quiet room on the neonatal unit. All study documentation was produced in English by the guiding researchers, and translated into Vietnamese by the local research team. Interviews were conducted in Vietnamese.

### Analysis

All interview transcripts were transcribed into English by the local research team prior to data analysis. Data was entered into the data management package NVivo v14 to facilitate analysis. Data were analysed using manifest content analysis, a method which focuses on the analysis of clearly evident components of the text in the interview data [14, 15]. Content analysis is a useful methodology when conducting exploratory work in a relatively unknown area, as it allows for the reporting and analysis of common issues in the data [16]. It also provides a useful method to quantify the data through counts of these issues [17, 18], allowing for comparison over a longitudinal study period. As this was the first research of its kind it Vietnam, from both the researchers and the participants perspectives, this approach was determined to be the most feasible in order to explore the research questions under investigation. Content analysis allows a systematic way of describing a phenomenon through the ability to distil words (text) into fewer content related categories, or themes [19, 20], with the aim of providing a new insight towards the research question through the development of these categories of description. At each time point, transcripts were read and re read to get a sense of immersion with the data (data was analysed between participants in each group separately (parents and nurses) at each phase of data collection). Initial coding of the data was then undertaken by the UK based researcher (KG) which involved recognising basic concepts within the text which reflected the research questions (meaning units of text). These codes were then grouped together, or condensed, into categories which reflected specific areas of discussion using descriptions as close to the text as possible. Frequency counts were assigned to each area to determine the commonality of the responses (in retrospection if discussed at T2 and not T1). The development of the codes and categories were discussed at each time point with the global research team to determine the credibility of the findings. Across the time points, the condensed units were compared and grouped into sub-themes representing the data. These sub-themes were finally placed into themes which represented the data as a whole, identifying separate areas of discussion which the participants discussed throughout the interviews (Table 1). The findings were again shared between the global research team to discuss any differences in opinion in categorisation and provide rigour to the study. This also provided an interesting approach to reflexivity in the study, as the analysis and interpretation were based upon different cultures and contexts. This minimised the risk of any individual biases towards the results and provided a depth of analysis which may not have been achieved otherwise.

**Table 1:** example of manifest content analysis

Code (meaning unit)	Condensed unit/category (frequency count at each time point)	Sub theme	Theme
“The relationship with the doctors and nurses is much better than before. Previously we have long distances and work under their command but after the training we become more intimate and interactive” (T2 N6)	Improved communication T1:0% T2:46% T3:81%	professional role	Perception of nursing role
“Some of the parents have improper manners with me because they are too worried about their children” (T3 N3)	Challenges in parent relationships T1:6% T2:38% T3:50%	Challenges to nursing role	

## Results

A total of 83 individuals participated in the study over 3 time points, including the same 16 nurses and 67 different parents at each time point (T1: 21 parents T2 & T3: 23 parents, respectively). As is typical for nursing in this setting, all participants were female. Results will be presented by group.

### Nursing participants

Amongst the participants there were 10 nurses and 6 midwives. Experience on the neonatal unit ranged from 2 to 30 years, with previous educational experience varied: 7 had completed education at ‘Trung Cap’ level (no equivalent in UK higher education), 8 at Cao-dang (equivalent to a certificate in higher education in the UK) and 1 at BSc level (equivalent to BSc in UK). Manifest content analysis of the transcripts identified 14 basic categories, which could be grouped into 3 themes: (1) perceptions of the role of the neonatal nurse, (2) perception of the parental role and (3) professional recollections. The first theme, perceptions of the neonatal nursing role, consisted of 5 subthemes: (1) nurses’ view of their professional role (2) nurses’ perceptions of their interactions with parents (3) nurses’ views of parental support for their role (4) nurses’ views of challenges to their role and (5) perceived ethical challenges. The theme, subthemes and corresponding verbatim quotes can be found in Table 2.

**Table 2:** Nursing interview analysis theme 1 and subthemes: perception of the neonatal nursing role

*Theme: (subtheme)*	*T1*	*T2*	*T3*
*Perception of role of neonatal nurse:*			
*Professional role*	“I get along with my colleagues well” (N2) “I take care of baby, supply the doctors’ order” (N15)	“The relationship with the doctors and nurses is much better than before. Previously we have long distances and work under their command but after the training we become more intimate and interactive” (N6)	“After the training course, the knowledge of nurses is raised much so they are more confident talking to doctors” (N1)
*Interaction with parents*	“I feel like I’m a bridge between babies and their parents, I tell the parents what the babies need, such as: when the baby need to breastfeed” (N6)	“We replace the parents to care for their babies and instruct them how to support us in the infant care” (N10)	“The relationship between nurses and parents plays an important role in caring for the babies. Nurses are the bridges to help parents understand the babies better because the babies are able to perceive everything around them” (N11)
*Parental support*	“Some parents are not nice but I can’t blame them, because they have small sick babies. I assure them and make them less nervous. The parent roles are very important, because they are the closest ones to the baby” (N3)	“We always sympathise with the patients parents because they are worried about their children. In any cases, I always behave gently and try to lessen their anxiety” (N3)	“We encourage parents to communicate with their children in order that they can develop more comprehensively and that will make their love more cohesive” (N13)
*Challenges to the nursing role*	“To me the challenge is sometimes I am not trusted by parents and they don’t listen to what I say and explain” (N9)	“Some parents are very intolerant, they do not understand and hear me explain the reasons for the non-positive situation of babies” (N8)	“Some of the parents have improper manners with me because they are too worried about their children” (N3)
*Ethical challenges* *(question changed from T1 – T2 so no data from T1)*		“My biggest challenge as a neonatal nurse is to inform parents that their babies died” (N10) T2: “I was extremely upset when we could not save babies’ lives” (N4)	“I was in a very awkward situation when I knew that we could not save the baby, but we still tried hard to win his life even though that would leave a legacy for him in the future” (N9)

Nurses increasingly reported a growing sense of collaboration with their medical colleagues through better communication after the training course (T1:0% T2:46% T3:81%). A similar number of nurses throughout the study (T1:62% T2:56% T3:56%) reported a facilitative role towards helping parents care for their infants through ways such as providing information, encouragement and empathy. There was an increase over time in nurses reporting challenges in their relationship with parents (T1:6% T2:38% T3:50%), mainly focused on communication issues*.* There was a small increase at T2 and T3 (*question modified from T1 so no data from T1)* in the number of nurses reporting ethical challenges (T2:12% T3:19%), for example when they could not save the life of a baby or when informing parents that their baby had died*.*

The second theme, nurses’ perception of the parents, consisted of 3 subthemes: (1) parental role (2) parental ward activities and responsibilities (3) parental participation in baby cares (Table 3).

**Table 3:** Nursing interview analysis theme 2 and subthemes: nurses perceptions of parents

*Theme (subtheme)*	*T1*	*T2*	*T3*
*Perceptions of parents:*			
*Parental role*	“Very important in caring the baby and help the baby’s condition become better” (N12) “If they know how to take care of the baby, it would be very helpful for the nurses” (N7)	“The role of the parents is very important here. I noticed that the babies who received loving care from their parents would recover sooner than the babies who their parents less concerned with” (N9) “Parents should pay attention to their babies condition more to inform the nurses, it helps to reduce the workload” (N11)	“The role of parents is very important. We need the presence of parents in time and support nurses in the care of their babies” (N2) “Parents should join their hands with nurses to take care of the babies” (N12)
*Parental ward activities & responsibilities*	“We guide the parents to keep the environment clean” (N5)	“The more important thing is that they should keep general hygiene in the patients room” (N1)	“Parents should…join sanitation activities around them because it will be to babies’ benefit” (N4)
*Parental participation in baby cares*	“They can take care of the baby, kangaroo care, breast feed. The babies who are with parents more will get better sooner” (N8)	“We just need the parents change diapers and make their babies feel more comfortable” (N14)	“Their [parents] duty is to make the babies feel more comfortable” (N2)

Nurses consistently reported that the parents played an important role in supporting their baby’s progress, although this decreased slightly as the study progressed (T1:56% T2:25% T3:38%). There was an increase over the study period in nurses reporting they felt that the role of the parents was to support the nurses through providing information about their baby or being present to take care of their baby (T1:0% T2:44% T3:50%)*.* Compared with T1, more nurses in T2 and T3 discussed that parents should adhere to hygiene principles on the neonatal unit (T1:6% T2:44% T3:44%), and that they should be involved in their infant’s care, such as bathing their baby, nappy changes, breastfeeding and making their baby feel comfortable.

The third theme, professional reflections, consisted of 5 subthemes: (1) pride as a neonatal nurse (2) working environment changes (3) perceptions of professional training (4) personal improvements and (5) advice for new neonatal nurses (Table 4).

**Table 4:** Nursing interview analysis theme 3 and subthemes: professional reflections

*Theme: (subtheme)*	*T1*	*T2*	*T3*
*Professional reflections:*			
*Sense of pride*	“I’m proud to have helped some babies overcome the death and get well” (N16) “I’m proud to see the facility is good, the nurses after being trained now work better, and the patients are being treated better” (N14)	“I feel proud to have contributed to the recovery of the babies” (N2) “[the] neonatal unit now have full equipment from sponsored projects, not lacking as before, therefore we have much more opportunities to treat patients” (N3)	“I feel proud to have contributed to the recovery of the babies and I feel happy for the babies to be discharged” (N7) “I think that the neonatal unit has changed both in awareness and in practice. I am proud to work here” (N5)
*Changes nurses would make about working environment*	“I am just a nurse I don’t want to change anything” (N2)	“We should review our mistakes and learn from that experience in order that we will not feel remorse for the mistakes we make” (N2)	“I look forward to passing on my knowledge and experience to junior nurses in order that they are able to care for newborn babies better” (N9)
*Perceptions of professional training*	“I gained a lot [of] benefits. I know more problems that need to be tackled. The newborns do have feeling for pain too, and now I listen to them, watch them carefully” (N11)	“The course has changed me a lot. Previously, I did not know the babies perception so I cared less about them. But after completing the training, I know more about their psychology and I know their feelings as well. I also know the joy, the sorrow, and the pain of the babies that rely on their faces. The more I talk to them, the more I love them” (N9)	“After the training I am thinking more about the psychology of the baby which can affect her treatment and prolong hospitalization” (N14)
*Perceptions of personal improvements*	T1: “Change me 100%, in both my perception and my practice” (N4)	T2: “I feel confident caring for newborn babies and am extremely grateful to all the teachers for that” (N16)	T3: “After the training, my clinical practice changed a lot. I feel more responsible for my work” (N13)
*Advice for new neonatal nurses*	“The most important part of a nurse’s work is caring” (N16) “You must concentrate on the work. The baby’s future depends on us. We give the baby a good future, or not?” (N9)	“I will remind new nurses that the newborn babies know the feeling hurt and they can feel everything around. Work with all your heart” (N13) “They should listen to the instructions from seniors and update new knowledge as much as possible” (N11)	“I advise them to treat the babies like their children to take better care” (N13) “I hope that all of the nurses should be trained more to improve their skills” (N10)

There was a large increase over time in nurses reporting that they took pride in seeing an infant’s condition improve (T1:0% T2:50% T3:69%). There was also a continuing increase over time in the number of nurses who reported that improved unit facilities made them feel proud (T1:0% T2:13% T3:25%)*,* although many remained unsure as to how they could improve their working environment. A small number of individual nurses suggested learning from mistakes, practical approaches to maximising efficiency of work time, and sharing knowledge amongst team members to improve the team process as a whole. Nurses increasingly shared the impact that increased knowledge from the professional training modules had upon their approach to the nursing environment in the neonatal unit, discussing various practices from positioning the baby, to noise and light exposure. When contemplating advice for new neonatal nurses, there was a small number of nurses at each time point who shared the importance of professional standards such as hygiene (T1:13% T2:9% T3: 9%) and continued learning and development (T1:19% T2:35% T3:22%), and a larger number who would share the importance of ‘caring’ for the babies (T1:30% T2:50% T3:50%).

### Parent participants

Manifest content analysis of the transcripts of parent interviews identified 14 basic categories of text, which could be grouped into 3 themes: (1) information sharing, (2) participation in care, and (3) personal experience.

The first theme, information sharing, comprised of 4 subthemes: (1) focus of discussion on admission (2) advice from professionals (3) updates about their baby’s condition and (4) limited information (Table 5).

**Table 5:** Parent interview analysis theme 1 and subthemes: information giving

*Theme: (subtheme)*	*T1*	*T2*	*T3*
*Information giving:*			
*Focus of discussion on admission*	“The doctor asked me about the condition of my baby, then I explained and she diagnosed the illness and told me about the treatment for my baby” (P1)	“We talked about congenital heart disease and mouth malformation of my baby” (P17)	“We exchanged a lot about her condition, and doctor don’t know what to say [sic], he had his upmost sympathy and concluded that the baby’s disease is difficult to treat” (P9)
*Advice from professionals*	“The nurses tell me about the baby too. When there’s something I want to ask, they are ready to answer” (P5)	“The doctors and nurses are very enthusiastic and give good advice to us” (P21)	“Doctor gave advice to me. Because my baby often vomits, doctor told me that I should hold her up while breastfeeding and keep still about 15 min after finishing” (P2)
*Updates about baby’s condition*	“Doctors and nurses keep me updated, they go to see me and the baby every morning and every night” (P5)	“Doctors go to see my son everyday and give me all the information I need about his condition” (P1)	“Doctors go to see the babies everyday and make us feel reassured about the condition of our baby” (P13)
*Limited information*	“I have to ask the nurses first to know about the baby’s condition. Nurse not tell me” (P9)	“I still do not understand well about the situation of my babies” (P10)	“I want to know more about my baby’s condition. I am afraid my baby may not be able to cope with the pain” (P1)

The majority of parents throughout the study consistently reported seeing a doctor and/or nurse on admission (T1:95% T2:87% T3:91%). When asked what the doctors and/or nurses discussed with them on admission, parents at all time points reported that they were informed about their baby’s condition, however there was a gradual decrease in this response as the study progressed (T1:95% T2:65% T3:57%). There was a gradual increase over time, however, of parents reporting that professionals offered advice or answered questions, potentially indicating more engagement and interaction with parents throughout the baby’s admission (T1:5% T2:13% T3:26%). The majority of parents consistently reported daily updates about their infant’s condition, which usually came from the doctors, although there was a slight decrease in this response as the study progressed (T1:95% T2:78% T3:74%). There was a small increase over the study period of parents reporting that they had as much information as they would like (T1:81% T2:83% T3:87%), however there was a small number of parents at time points 2 and 3 (3 parents [13%] at each respectively) who reported they did not have as much information as they would have liked about their baby’s condition*.*

The second theme, participation in care, comprised of 3 subthemes: (1) perceived engagement (2) desired involvement (3) perceptions of the nursing role (Table 6).

**Table 6:** Parent interview analysis theme 2 and subthemes: participation in care

*Theme: (subtheme)*	*T1*	*T2*	*T3*
*Participation in care:*			
*Perceived engagement*	“When I didn’t know how I massage the baby, they showed me. They care about me and my baby a lot” (P15)	“The nurses taught me how to breastfeed. Thanks to the good care of nurses, my baby is growing stronger” (P4)	“The nurses create all favorable conditions for me to take good care of my baby” (P18)
*Desired involvement*	“I want to learn from doctor and nurses to take good care of my baby” (P15) “It would be great if there is presence of the mom when a baby is being treated, because mom has the breastmilk for baby. If I can get involved in the baby’s treatment, I can somehow help reducing the nurses’ work and my baby not cry so much” (P1)	“I want to do anything I can to make the baby get well soon” (P5) “I want to be actively involved in treatment for him” (P20)	“The role of the parents is very important. I also need fully updated information and regularly talk with the nurses and doctors on the neonatal unit” (P2) “I wish to care for my baby myself, it sets my mind at rest” (P6)
*Perceptions of the nursing role*	“The nurses here are really doing a good job. They give me much information about the baby’s condition, about when to leave the hospital. At night they come to check the baby an remind me of something if I forget to do while sleeping” (P6)	“The role of the nurse is very important. The nurse gave many advices and methods of taking good care of the baby” (P16)	“The role of the nurse in the care of the baby is very important. The nurse gave many good advice for example the baby should be breastfed entirely, should not be bottle fed and when the baby could not poo 3 days after bottle-feeding, she reminded me frequently and helped the baby to stool. It made her sleep well” (P3)

Despite a small decrease over time, the majority of parents throughout the study reported feeling engaged during their infant’s stay, and that they felt their role was one of care-giver to their baby (T1:81% T2:74% T3:74%). The number of parents wanting more involvement in their baby’s care increased from T1 to T2 but remained unchanged between T2 and T3, reflected in parents discussing how they would like more involvement in activities such as kangaroo care and more practical advice about how to care for their baby (T1:0% T2:26% T3:26%). For many parents, however they were unsure about how they could become more involved. Over the study period there was an increase in parents sharing positive feedback for the nursing team (T1:29% T2:43% T3:48%), potentially resulting from the frequency that parents reported nurses provided information about their baby to them (T1:0% T2:13% T3:35%).

The third theme, personal experiences, comprised of 3 categories: (1) positive experiences (2) practical challenges and (3) changes to benefit parents in the future (Table 7).

**Table 7:** Parent interview analysis theme 3 and subthemes: personal experiences

*Theme (subtheme)*	*T1*	*T2*	*T3*
*Personal experiences:*			
*Positive experiences*	“I don’t have anything ask more and nothing to complain” (P21)	“The neonatal unit met the best necessary conditions for my child’s recovery and comply with hygienic regulations. I am really satisfied when being treated here” (P16)	“The quality of service is very good, so I want all babies are treated here” (P14)
*Practical challenges*	“I think the nurses should go to check me and the baby more often long time no nurse come to me” (P3)	“I think there should be a second person in the room to help the mom taking care of her baby” (P15)	“I would like to consult with doctors about his situation much more but I have no opportunity to do it” (P8)
*Changes for future parents*	“They should be more clean many parents dirty and use mobile phone and noise the doors” (P10)	“Some parents close or open the door so hard that make the babies wake up and annoy the others It would be better if there is a sliding door” (P2)	“The mothers should have a wash before contacting with their babies” (P8)

There was an overall increase of parents reporting satisfaction with their baby’s care despite an initial fall at T2, with parents citing unit cleanliness, the kindness of staff and assistance with caring for their baby (T1:48% T2:30% T3:57%). At the same time, however, parents increasingly reported practical challenges which they faced such as inflexible ward visiting schedules, staff shortages limiting nurses’ ability to help them care for their baby, or physicians’ opportunities to talk with them (T1:33% T2:48% T3:61%) highlighting that there are areas which still require attention to improve overall parental satisfaction. A small number of parents at each time point discussed concerns with other parents on the neonatal unit such as a lack of cleanliness or being too noisy, and their concerns about the impact of these upon their own infants and others (5, 1 and 1 parents respectively). When parents were asked whether there was anything that they would change for future parents coming into the neonatal unit, the majority of parents were unsure (T1:24% T2:52% T3:57%) however individuals at each time point did suggest actions such as more nurses to help mothers care for the babies, better information sharing about the babies condition and more equipment.

## Discussion

The aim of this study was to explore changes in the perceptions and attitudes of nurses and parents towards their experiences in the neonatal unit following a neonatal nursing education intervention in a single neonatal unit in central Vietnam. The study has some limitations. As interview methodology remains a fairly new concept in Vietnamese culture, our approach encountered some cultural differences which meant we did not always achieve the depth of information in our responses that we had envisaged, despite piloting the interview schedules. Demographic information was also not collected from parents, making it difficult to explore whether their experience may have been impacted by previous experience or length of stay. It is also important to note parent participants interacted with nurses who had taken part in the study alongside with a number who had not. Our parental findings are therefore based on their overall experience on the neonatal unit. What we have shown, however, is that neonatal qualitative research in middle income countries can be achieved through international partnership. The results of our interviews highlight many interesting changes in the responses over three time points. For nurses, this included changes in their perception of the nursing and parental role, and a growing awareness of ethical issues and challenges communicating sensitive information with parents and professional standards. For parents these changes included a growing sense of wanting more participation in their infants’ care through information sharing about their baby’s condition, practical advice around baby care, a growing awareness of ward practices which affect their engagement and satisfaction with care, and an increase in positive feedback for nurses.

These results provide insight into the culture of neonatal nursing in one unit in central Vietnam, and its continuing practice changes following an educational intervention. For nursing staff, a growing sense of professional identity can be seen through their increased confidence to interact with the medical team, their support of parents, and their pride in the care they provide and the unit in which they work. Whilst a growing number of nurses reported that parents should support them in their work, this could reflect an increase in awareness of the extent of their nursing role in developmental care, pain management, and symptom recognition and management. It may also be reflective of a growing belief in the ability of parents to provide care for their own infant during admission. For parents, the gradual increase in wanting more involvement in their infant’s care may reflect the nurses’ perceived expanding role. Parents wanting and potentially gaining more involvement in their infants’ care could promote awareness of practicalities which hinder their ability to do so. The difficulties in communication with parents that nurses reported is consistent with the parental responses that they gain most of the information about their baby’s condition from the doctors along with the decrease in reported information sharing and updates. An area for future training may therefore include communication skills training for nurses on how to communicate effectively with parents, thereby improving both nursing and parental experience.

Very few studies have performed qualitative research in this area; however one study in particular carried out qualitative interviews with 198 parents of infants who were hospitalised within the first month of life in Cambodia, Malaysia, Laos and Vietnam, and explored perceived barriers to newborn care [21]. Results highlighted that parents had low levels of satisfaction with their neonatal experiences, with parents from one unit in Hanoi, Vietnam, specifically (*n* = 48) citing issues such as availability of medications, privacy, cleanliness and staff demeanour as areas which negatively impacted their experience. A small number of parents in our study highlighted hygiene as an issue affecting their satisfaction, although this was often directed towards other parents on the unit rather than the unit itself. The positive appraisal of staff in our own study is in contrast with the results from Martinez et al. This could be attributed to limitations in that the infants of the parents interviewed were still inpatients in our study, and therefore the parents may have felt uncomfortable criticising the providers of their infants care; alternately, it could reflect a change in the attitudes of the nursing personnel towards parents following training in family-centred care. Martinez et al. also conducted surveys with 212 health care providers, exploring their perceptions of barriers in providing care. The surveys undertaken with health care providers in Vietnam (*n* = 63) highlighted a lack of nursing staff as a concern to providing more aggressive treatment on the neonatal unit [21]. While this was reported by nurses in our study, we also found that parents felt that there should be more staff to help mothers care for their infant. Reports by the nurses that parents should help support them to care for the infants also suggests a need for more nursing support on the neonatal unit. Health care providers cited a lack of staff training as impacting the provision of neonatal care in the study by Martinez et al. [21], which is emphasised by our own study in which the nurse highlight their sense of pride from their training through increased knowledge, impact upon practice and improved infant care. This suggests that implementing an educational intervention can build staff confidence, lead to an increased sense of professional identity, and help nursing staff to perform their role. A systematic review of motivation and retention of health workers in developing countries found that career development, educational opportunities and recognition from managers were major motivational themes [22]. The Human Development Report from the United Nations suggests better job opportunities through education and development could result in a more engaged workforce who are more efficient, helping in turn to maintain and raise the standards of their care as a source of pride [23]. This area requires further research to determine whether a more educated and motivated nursing workforce may facilitate a reduction in infant mortality.

The majority of research undertaken in the neonatal context in Vietnam focuses on causes of infant mortality and morbidity [4, 5, 24, 25], implementation and outcomes of community-based interventions [8, 26, 27] and the knowledge base of health care staff [6, 7]. The results from these studies collectively suggest that knowledge transfer and resulting practice change may be hugely important factors in reducing infant mortality. Identification of areas of knowledge improvement such as infection control, symptom recognition and temperature control are highlighted across many of the studies. Whilst our study does not directly assess the knowledge of the staff, it suggests an increasing sense of professionalism accompanying an expanding understanding of the extent of the neonatal nursing role. Further research is therefore required with the nurses in our own study group to determine the degree of knowledge acquisition, and whether the content of the educational training impacts upon their attitudes and opinions of neonatal care. Similarly assessment of retention of knowledge is critical, as decreasing competence over time is a concern [28]. In order to effect changes in practice, teaching and learning needs to be maintained over a sustained period of time to ensure clinical currency. Recent studies have shown that this is feasible and successful. Thukral et al. evaluated an educational programme with newborn care providers in resource limited settings in India and Kenya through satisfaction surveys, multiple choice examinations, objective structured clinical exams (OSCEs) and confidence assessments [29]. Shrestha et al. successfully examined changes in knowledge and practice after a care of the newborn intervention with nurses in maternity, emergency and delivery units in India [28]. Development of effective baseline and outcome measures is required to formally assess the nurses in our own study for future intakes of staff into the education programme, and if the interventional model is shared with other local hospitals.

Despite the lack of research into parental experiences in neonatal care in Vietnam and surrounding similar countries, the results from our study suggest that the needs of parents who have an infant in the neonatal unit are universal and not impacted by the country or it’s economic standing. Parents reporting a desire for more information and more participation reflect interview studies with parents from many developed countries such as the UK, USA and Australia; [30–33] the main difference being that parent outcomes are arguably better defined, measured and studied in developed countries. Further research into parental experiences and outcomes (such as stress, anxiety, post-traumatic stress, depression) may help us to understand more about the parental experience and how to support them in countries such as Vietnam. This may ultimately impact upon infant mortality and morbidity, as negative prior experience of neonatal care served as a barrier to parents accessing care for future infants in the study by Martinez et al. [21] More positive parental experience may therefore result in parents being more willing to access neonatal care for subsequent babies, potentially helping to improve infant outcomes in developing countries.

## Conclusion

This study has highlighted that the introduction of an educational intervention can produce short term effects upon both nursing and parental experience of neonatal care in one unit in central Vietnam. Further research is required to determine the long term impact of the intervention, the ability of nurses to translate knowledge into clinical practice through assessment of nursing knowledge and competence, and the impact and needs of parents. Understanding more about these factors will allow us to continue to improve the experiences of parents and nurses and highlight how these areas may contribute towards the reduction of infant mortality and morbidity in Vietnam.

## Additional files

Additional file 1: Interview schedule for neonatal nurses. (DOCX 484 kb)

Additional file 2: Interview schedule for parents. (DOCX 483 kb)
