# Supplementary material for: Nursing & parental perceptions of neonatal care in Central Vietnam: a longitudinal qualitative study
Source: BMC Pediatr. 2017 Jul 11;17:161. doi: 10.1186/s12887-017-0909-6 (PMC5505145; doi:10.1186/s12887-017-0909-6)
Supplement: Supplementary file 1 — Interview schedule for neonatal nurses. (DOCX 484 kb) [file 12887_2017_909_MOESM1_ESM.docx]

Thank you for agreeing to take part in the final interview in this study. The format of today’s interview is similar to the others, and is designed to get an understanding of your thoughts about your role as a nurse on the neonatal unit. Some of the questions will be the same as before so we can see whether your thoughts have changed since we started the study. We would like you to feel completely comfortable talking to us as we assure you that your responses will remain completely anonymous. With your permission, we will digitally record the interview however nothing that is said in the interview will be linked to you personally.

The interview is very informal and is designed to last about 30 minutes, however please feel free to take your time. If you would like to stop the interview at any point, please just say, you do not have to give a reason. Do you have any questions before we begin?

We would like to understand a little bit about how you see yourself as a nurse. How would you describe your role on the neonatal unit?

- How do you see your relationship with the doctors?
- What do you think is your role in relation to the parents?

What is important to you as a neonatal nurse?

- What do you take pride in?
- Is there anything that you would change (good or bad) about your role?
- What is your biggest challenge as a neonatal nurse?
- Have you encountered any situations which you personally found ethically / morally difficult on the neonatal unit? If yes, could you tell us a little bit about the situation and why you found it difficult?

We would like to understand about what you think of the parents you work with.

- How do you see the role of parents on the neonatal unit?
- What sort of activities on the neonatal unit do you think the parents should be able to get involved in? (such as ward rounds, bathing, kangaroo care etc)

Now I would like to know about your perceptions of the training you have received as part of your job as a neonatal nurse.

- Do you feel that the training has changed your practice in any way? In what ways?
- What are your thoughts about the training now you have been qualified for over a year?
- Now you have more experience, would you change anything about the training you received?

Now I would like to ask whether you feel you have changed as a neonatal nurse during the time in the study.

- In what ways do you think you have changed?

And finally, my last question is around your thoughts for future neonatal nurses. If you met a new neonatal nurse who was joining your team tomorrow, what is the most important thing you would want to tell them about working on your neonatal unit as a nurse?

Thank you so much for taking the time to talk with me today and helping in our study. Is there anything that you would like to add that we haven’t covered? Thank you once again.
