# Supplementary material for: Nursing & parental perceptions of neonatal care in Central Vietnam: a longitudinal qualitative study
Source: BMC Pediatr. 2017 Jul 11;17:161. doi: 10.1186/s12887-017-0909-6 (PMC5505145; doi:10.1186/s12887-017-0909-6)
Supplement: Supplementary file 2 — Interview schedule for parents. (DOCX 483 kb) [file 12887_2017_909_MOESM2_ESM.docx]

Interview Schedule

Thank you for agreeing to take part in this interview. The interview is designed to get an understanding of your experiences during your infant’s time in the neonatal unit, and we would like you to feel completely comfortable talking to us as we assure you that your responses will remain completely anonymous. With your permission, we will digitally record the interview however nothing that is said in the interview will be linked to you personally.

The interview is very informal and is designed to last about 30 minutes, however please feel free to take your time. If you would like to stop the interview at any point, please just say, you do not have to give a reason. Do you have any questions before we begin?

1. To start, please tell me a little bit about why your baby has been admitted to the neonatal unit?

1a. When you first arrived on the neonatal unit, who did you meet?

1b. Did you talk to any of the doctors or nurses?

1b. What kind of things did they talk to you about?

1c. Were you given the parent admission letter and informed about the work of the neonatal unit?

1. And how are you kept up to date with (baby’s name) condition on the neonatal unit?

2a. Who keeps you updated? How often are you updated?

2b. Do the nurses talk to you when you visit about how (name) is doing or do you have to ask?

2c. Do you always have as much information as you would like about how (name) is progressing?

1. Do you feel involved in (name’s) treatment and care? How do you see your involvement?
2. We would like to know how you see the role of the nurse in caring for (name). What sort of things do you think the nurses do to take care of your baby?

4a. Is there anything that you would like to see the nurses do more / less of?

1. And now we would like to ask you about how your role on the neonatal unit.

5a. What do you expect to get involved in whilst you are on the neonatal unit?

5b. Is there anything that you particularly enjoy / do not enjoy about being a parent on the

neonatal unit?

1. What information have staff given you to get you ready to take (name) home?

7. My last question is about your overall experience on the neonatal unit. From your experience, is there anything, good or bad, that you would change for future parents coming onto the unit?

7a. Why is this?

Thank you so much for taking the time to talk with me today. Is there anything that you would like to add that we haven’t covered? Thank you once again.
